# Low temperature CO oxidation by doped cerium oxide electrospun fibers

**DOI:** 10.1186/s40580-020-00234-7

**Published:** 2020-06-29

**Authors:** Myeongseok Sim, Buhua Wang, Tae-Sik Oh

**Affiliations:** 1 Department of Chemical Engineering, Auburn University, Auburn, AL 36849 USA; 2 Department of Chemistry and Biochemistry, Auburn University, Auburn, AL 36849 USA

**Keywords:** CO oxidation, Electrospinning, Cerium oxide, Metal-free

## Abstract

We investigated CO oxidation behavior of doped cerium oxide fibers. Electrospinning technique was used to fabricate the inorganic fibers after burning off polymer component at 600 °C in air. Cu, Ni, Co, Mn, Fe, and La were doped at 10 and 30 mol% by dissolving metal salts into the polymeric electrospinning solution. 10 mol% Cu-doped ceria fiber showed excellent catalytic activity for low temperature CO oxidation with 50% CO conversion at just 52 °C. This 10 mol% Cu-doped sample showed unexpected regeneration behavior under simple ambient air annealing at 400 °C. From the CO oxidation behavior of the 12 samples, we conclude that absolute oxygen vacancy concentration estimated by Raman spectroscopy is not a good indicator for low temperature CO oxidation catalysts unless extra care is taken such that the Raman signal reflects oxide surface status. The experimental trend over the six dopants showed limited agreement with theoretically calculated oxygen vacancy formation energy in the literature.

## Introduction

CO oxidation is a representative model oxidation reaction in heterogeneous catalysis [1]. When studying model catalysts such as three-phase boundary (TPB) controlled platinum-YSZ (yttria stabilized zirconia) or single crystal platinum, CO oxidation was chosen to be the reaction of interest [2, 3]. CO is a well-known environmental pollutant, and the molecule strongly binds to the surface of precious metal catalysts blocking other desired reactants from the available surface sites [4]. CO oxidation is a one way to mitigate these problems. It is desired to have the oxidation occurring at as low temperature as possible to reduce the energy cost and minimize air pollution.

Nanostructuring is essential to have highly active catalyst [5, 6]. The most common fabrication route for oxide nanoparticles starts from salts such as nitrates, acetates, and sulfates followed by conversion to oxide using thermal treatment. In many cases, sacrificial templates were used to achieve nanostructuring. Electrospinning is one such template-based strategy where fibrous polymer backbone structure leaves ceramic fibers after thermally removed [7–10]. In this process, metal salts are firstly dissolved in a polymeric solution. After spinning the polymer fiber through a metal tip under electrical field, the carbonaceous component in the resultant fiber mat gets burnt away in air. The remaining oxide phase loses long-range connection due to its brittleness; however, the high aspect ratio is still intact. This porous structure with high surface area is attractive for many catalysis applications. For instance, electrospun TiO_2_ fiber was used to support Pd-Au nanoparticles for NO decomposition by CO [9], and electrospun cerium oxide fiber was used to support Pd-Cu nanoparticles for water–gas shift reaction [10]. Apart from catalysis, it is demonstrated that the electrospun copper oxide fiber can be reduced to copper fiber for transparent electrode application [11]. Going beyond simple binary oxides, La_0.6_Sr_0.4_Co_0.2_Fe_0.8_O_3-δ_ perovskite oxide fiber was utilized to fabricate a solid oxide fuel cell air electrode [12].

In addition to lowering energy cost by low temperature operation, catalysts free from precious metals are attractive due to low materials cost. Metal-free oxide system is expected to suffer less from coarsening over long term operation due to higher thermal stability than metals. Self-supported oxide can be ideal if it can give high enough conversion rate at the target temperature. It is logical to start from a common support oxide. We can find many different oxides that have been used to support metal catalysts. Among them, we chose cerium oxide. Cerium oxide is an interesting material that has shown synergistic impact with metallic nanoparticles for volatile organic compound oxidation [13, 14].

Cerium oxide is a well-known oxygen storage material that is used in three-way catalytic converters and solid oxide fuel cell fuel electrodes [15–20]. The easy transition between cerium cation oxidation states (4+/3+) and the formation of oxygen vacancies give superior redox property in ceria [21–25]. Furthermore, undoped nanoscale CeO_2_ displays significant catalytic activity compared to bulk undoped CeO_2_ due to increased specific surface area and easier oxygen vacancy formation [26, 27].

In this study, ceria-based nanofibers were synthesized using the electrospinning technique to investigate their catalytic activity toward CO oxidation. No precious metal particles were involved in this study. Doping inexpensive metals has been proven effective in many studies [28–31]. To study the effects of dopants, Cu, Ni, Co, Mn, Fe, and La were doped at 10 and 30 mol% by dissolving metal salts into the polymeric electrospinning solution. This work demonstrates the potential of electrospun self-supported cerium oxide catalyst for CO oxidation.

## Experimental

### Reagents

Cerium nitrate hexahydrate (Alfa Aesar), polyvinylpyrrolidone (PVP, MW 1,300,000, Alfa Aesar), and *N*′,*N*-dimethylformamide (DMF, VWR Life Science) were used to produce undoped ceria nanofibers. Copper acetate monohydrate (Sigma Aldrich), nickel nitrate hexahydrate, iron nitrate nonahydrate, cobalt nitrate hexahydrate, manganese nitrate tetrahydrate, and lanthanum nitrate hexahydrate (all from Alfa Aesar) were used for doping the oxide. All reagents were used without any further purification.

### Preparation of the electrospinning solution

Metal salts were dissolved in DMF along with PVP to make the polymeric solution. The high molecular weight of PVP is crucial for stable jet formation under electrical field. Solutions with 10 and 30 mol% of metal dopants were first prepared from appropriate amounts of metal salts and 1.93 g cerium nitrate hexahydrate in 15 mL of DMF. After complete dissolution of metal salts, 1.93 g of PVP was added to the solution. The solution was then left under magnetic bar stirring overnight at room temperature to produce a viscous and homogeneous solution.

### Electrospinning

The final solution was loaded in a BD 10 ml plastic syringe equipped with a stainless-steel blunt-tip needle (21 gauges, 0.51 mm inner diameter). The syringe was mounted horizontally and fastened to a syringe pump, and the flow rate of the pump was set to be 1.2 ml h^−1^. The fiber was collected on aluminum foil with the tip-to-plate distance at 15 cm. The applied voltage was 18 kV, producing 1.2 kV cm^−1^ electric field. Under this electrospinning condition, a uniform fibrous structure was formed. During the process, the relative humidity and temperature were maintained below 40% and at room temperature, respectively. When the relative humidity gets higher than 40%, it becomes impossible to attain uniform fiber from the polymeric solution. After collecting the fiber from 600 cm [2] central area on the aluminum foil, the electrospun fiber was later calcined at 600 °C for 3 h or 6 h with a heating rate of 1 °C min^−1^ in the air to burn off PVP and obtain oxide nanofibers. Fiber outside the 600 cm^2^ central region had non-uniform texture, so it was not calcined for further use. After the burning off the polymer, extra care was taken when handling the remaining material due to its extreme lightness. The samples were named after the dopant element and doping level. Cu10 means Ce_0.9_Cu_0.1_O_2-δ_ fiber, for instance.

### Characterization of metal oxide nanofibers

Zeiss EM10 transmission electron microscope (TEM) were employed to study morphology and the size of the pure ceria nanofibers after calcination. The TEM was operated at 60 kV. To check the oxide phases, X-ray diffraction patterns were collected with Cu Kα radiation in Bragg–Brentano geometry (PROTO AXRD, 40 kV, 30 mA). TriStar II 3020 was employed to measure the BET surface area of oxide fibers. The fiber sample was loaded to the adsorption cell after pressing a pellet and breaking it into pieces. This is to be consistent with the CO oxidation test condition. The nitrogen physisorption was performed at − 196 °C after degassing samples at 350 °C for 5 h under nitrogen flow to eliminate water and other contaminants. Raman spectroscopy (inVia, Renishaw) was used to analyze the structure of the catalysts. The instrument used 514.5 nm laser and the measurement range was 200 to 800 cm^−1^.

### Catalytic testing

**Fig. 1:**
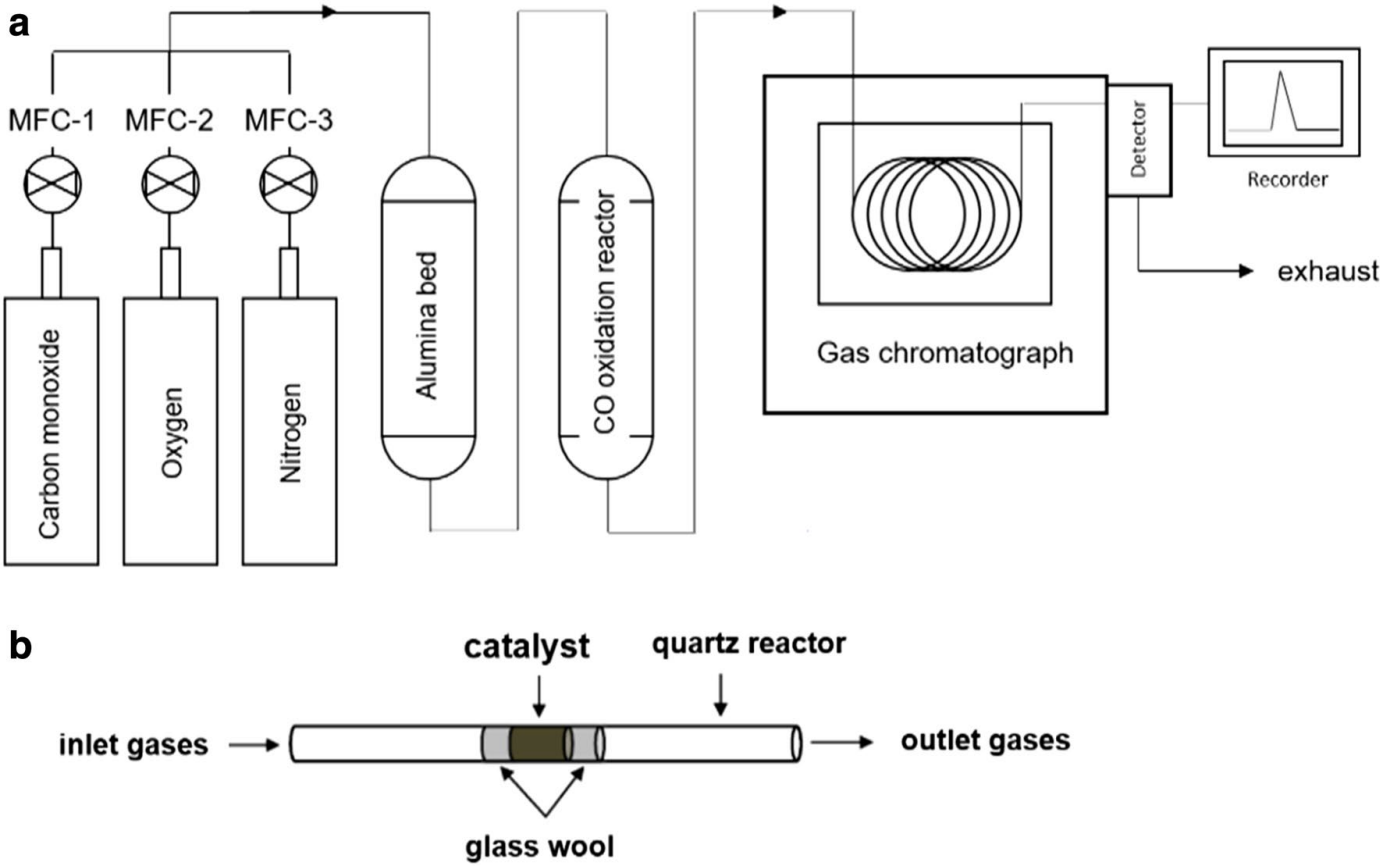
**a** CO oxidation reactor setup. The temperature for alumina hot bed was 400 °C. **b** The CO oxidation reactor. 0.2 g of the sample got packed in 1 cm length space in the 4 mm I. D. quartz tube. The same quartz tube was used for alumina bed also

The CO oxidation experiments were performed in a quartz tubular reactor (I. D. = 4 mm). A pressed pellet (0.2 g) made from electrospun fiber was broken into pieces to be loaded in the reactor. The catalyst sample was sandwiched by glass wool stubs placed 1 cm apart. The feed gas had 1 vol% CO and 2 vol% oxygen in balance nitrogen. The flow rates were controlled by three mass flow controllers assigned for each gas. The total flow rate was fixed to be 100 sccm (WHSV, weight hourly space velocity = 30,000 mL h^−1^ g^−1^). After the three mass flow controllers the feed gas mixture went through a hot alumina bed to first thermally decompose iron pentacarbonyl coming from the CO gas tank before it reaches the CO oxidation reactor. The iron pentacarbonyl is hard to avoid since the molecules form from the reaction at ambient temperature between the pressurized gas and the cylinder wall material [32]. The temperature of the alumina bed was set at 400 °C. 0.4 g of high surface area alumina powder (Alfa Aesar, stock number 43855) was uniformly packed in the quartz tube (I. D. = 4 mm, bed length = 30 cm). Without the hot alumina bed, there was a reddish color change to the cerium oxide samples. When the alumina hot bed was in place, there was no color change to the cerium oxide samples. CO oxidation after the alumina bed was not detected ensuring that all the CO conversion is exclusively from the doped/undoped cerium oxide electrospun fiber samples. The CO oxidation experiments were carried out from room temperature to the temperature of 100% conversion without any separate pretreatment. This complete conversion temperature varies based on what catalyst sample is in action. At every measurement temperature, the fiber catalyst sample was exposed to input flow for 30 min before gas sampling rather than continuous gas sampling with continuous temperature ramp. The gas composition was analyzed using a gas chromatograph (SRI 8610C) with a thermal conductivity detector. Carboxen-1000 column was used to separate CO and CO_2_. Nitrogen was the carrier gas for the gas chromatograph. Gas conversion was calculated from the areas of CO and CO_2_ peaks. CO_2_ generation rate (mol g^−1^ s^−1^) was calculated from conversion, input CO concentration, total volumetric flow rate, and fiber mass. For Cu10 sample, the best catalyst in this work, the CO oxidation experiment was repeated three times to check the catalyst stability. After the first run, the sample was cooled down in nitrogen from the 100% conversion temperature. The second run started again from room temperature to 100% conversion temperature. After cooling in nitrogen again, the sample was heated in air to 400 °C and held for 1 h. The heating and cooling rate was 1 °C/min for this treatment. The final third run was carried out. For all CO oxidation runs, the same condition was used throughout. The experimental setup is illustrated in Fig. 1.

## Results and discussion

### Characterization of electrospun fibers

**Fig. 2:**
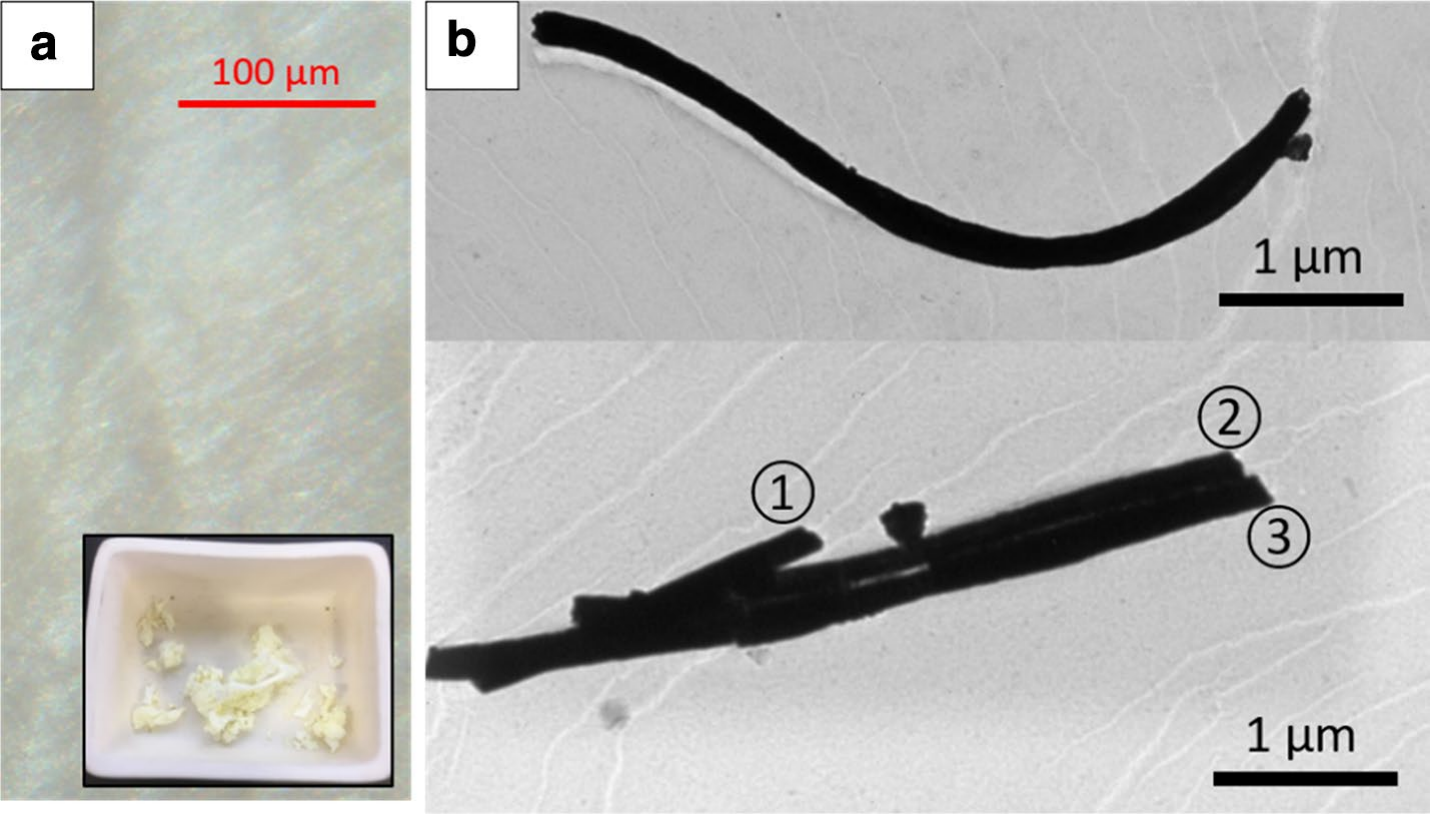
**a** An optical image of as-spun fiber mat. Inset shows the oxide fiber right after burning off PVP. Typical yellowish cerium oxide color can be seen. **b** TEM images from the undoped cerium oxide fibers

After burning off the polymeric constituent at 600 °C in air for 3 h, the remaining oxide fiber was examined by TEM. Due to brittleness, the length of individual oxide fiber is limited. Figure 2a shows an optical image of an as-spun fiber mat containing cerium source only. A uniform texture can be confirmed by optical microscopy. The inset shows the resulting cerium oxide fiber still in the alumina boat that is used for burning off PVP. Extreme care needs to be taken when handling this cerium oxide fiber since it is very light. It easily becomes airborne with small mechanical agitation. After dispersing the cerium oxide fiber in ethanol, we drop-coated a copper TEM grid for morphology observation. Figure 2b shows undoped cerium oxide fibers. The top image is from one fiber, and the bottom image shows three fibers fused together. The average diameter of the fibers in Fig. 2b is about 200 nm. In this work, we did not try to reduce this fiber diameter since the focus is more on the effect of dopants. The BET surface area of fibers, inversely proportional to the diameter of fiber, ranged from 50 to 100 m^2^/g suggesting that the diameter varies within the factor of 2.

**Fig. 3:**
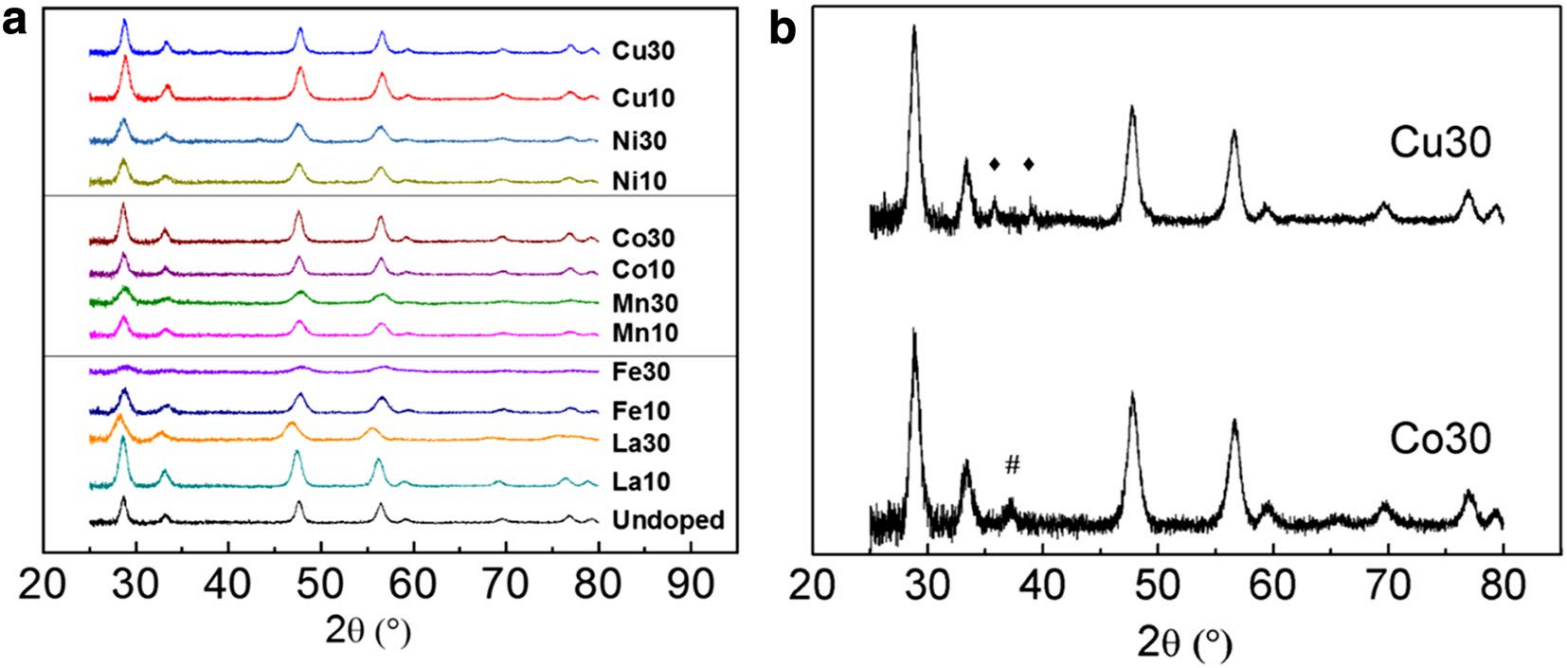
**a** XRD patterns from as-calcined fiber samples (600 °C, 3 h). **b** Zoomed-in XRD patterns of Cu30 and Co30 to show second phase peaks. Closed diamond show CuO peaks, and hash marks show Co_3_O_4_ peaks. For all other samples, no second phase peaks were detected when calcined at 600 °C for 3 h

For all fiber sample variations, fluorite structure was confirmed by X-ray diffraction (XRD). Figure 3a shows doped cerium oxide diffraction patterns after 3 h calcination at 600 °C. For 30 mol% doping of Cu and Co, CuO and Co_3_O_4_ second phase peaks were detected respectively since their doping level is apparently more than the solubility limit in the cerium oxide lattice. For 30 mol% Ni doped fibers, NiO peaks were detected only when calcination holds for 6 h as shown later.

### CO oxidation activity

**Fig. 4:**
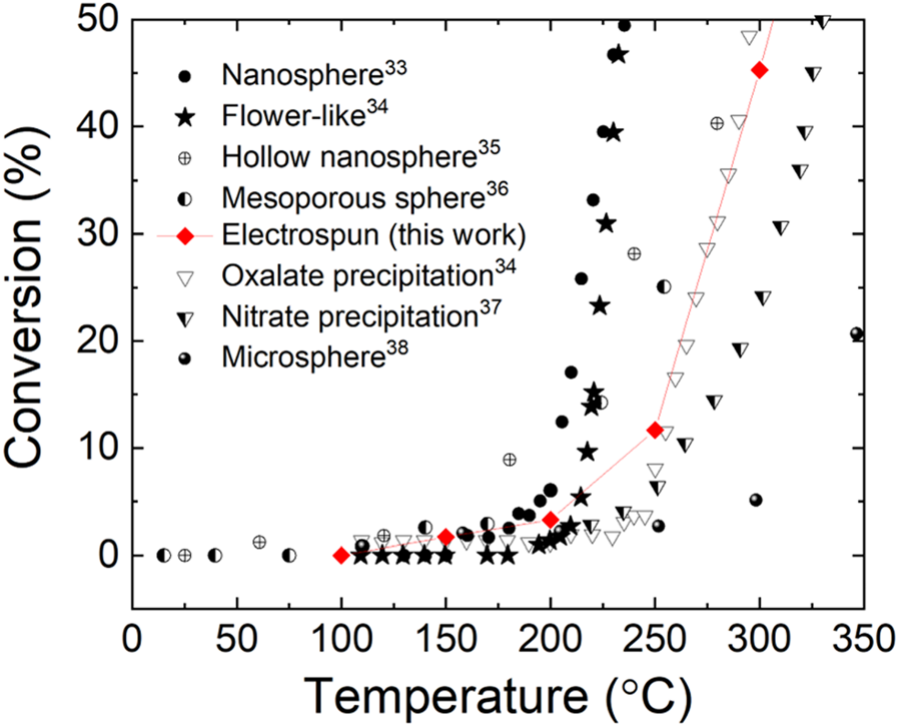
CO conversion from nanostructured, undoped ceria samples

We firstly compared CO oxidation behavior of our undoped ceria electrospun fiber to other undoped ceria catalysts reported the literature. In all cases, hydrogen was not in the feed gas. Figure 4 shows CO conversion comparison [33–38]. The conversion curves indicate that the current electrospinning-firing (600 °C, 3 h) process, as expected form the high calcination temperature, was not as efficient as nanosphere fabrication [33] or flower-like structure formation [34] in terms of nanostructuring. However, it is clearly more effective than precipitating from nitrate source (500 °C, 5 h calcination) [37]. The low temperature CO conversion behavior of the undoped electrospun ceria catalyst is very similar to the one from oxalate precipitation (300 °C, 1 h calcination) [34]. The necessity of high temperature to burn off polymer imposes limitation in nanostructuring for electrospun fibers. Using the same polymer and 600 °C calcination temperature, the surface area never surpassed 150 m^2^/g for any oxide fiber we made. We did not lower the polymer burn-off temperature less than 600 °C in this work to make sure there is no residual carbon from PVP. Table 1 summarizes the CO oxidation conditions and CO_2_ generation rate at 200 °C for the undoped samples shown in Fig. 4. The CO_2_ generation rate at 200 °C for our electrospun fiber sample is within the reported range from other nanostructured, undoped ceria catalysts.

**Table 1:** CO oxidation conditions for nanostructured, undoped ceria samples

Fabrication method	Feed gas	Balance gas	Highest temperature and duration before CO oxidation	WHSV (mL h^−1^ g^−1^)	CO_2_ generation rate at 200 °C (mol g^−1^ s^−1^)
Autoclave heating [33]	1% CO, 10% O_2_	N_2_	300 °C, 1 h	96,000	6.5E−07
Autoclave heating [34]	1% CO, 10% O_2_	N_2_	150 °C, 5 h	96,000	1.4E−07
Autoclave heating [35]	1% CO, 20% O_2_	N_2_	350 °C, 1 h	12,000	2.1E−07
Autoclave heating [36]	1% CO, 16% O_2_	N_2_	180 °C, 200 m	30,000	2.0E−07
Electrospinning (this work)	1% CO, 2% O_2_	N_2_	600 °C, 3 h	30,000	1.1E−07
Oxalate precipitation [34]	1% CO, 10% O_2_	N_2_	300 °C, 1 h	96,000	1.3E−07
Nitrate precipitation [37]	9.98% CO, 10.2% O_2_	Ar	500 °C, 5 h	33,000	4.5E−07
Microsphere templating [38]	0.24% CO, 15% O_2_	Ar	600 °C, 12 h	60,000	3.6E−08

**Fig. 5:**
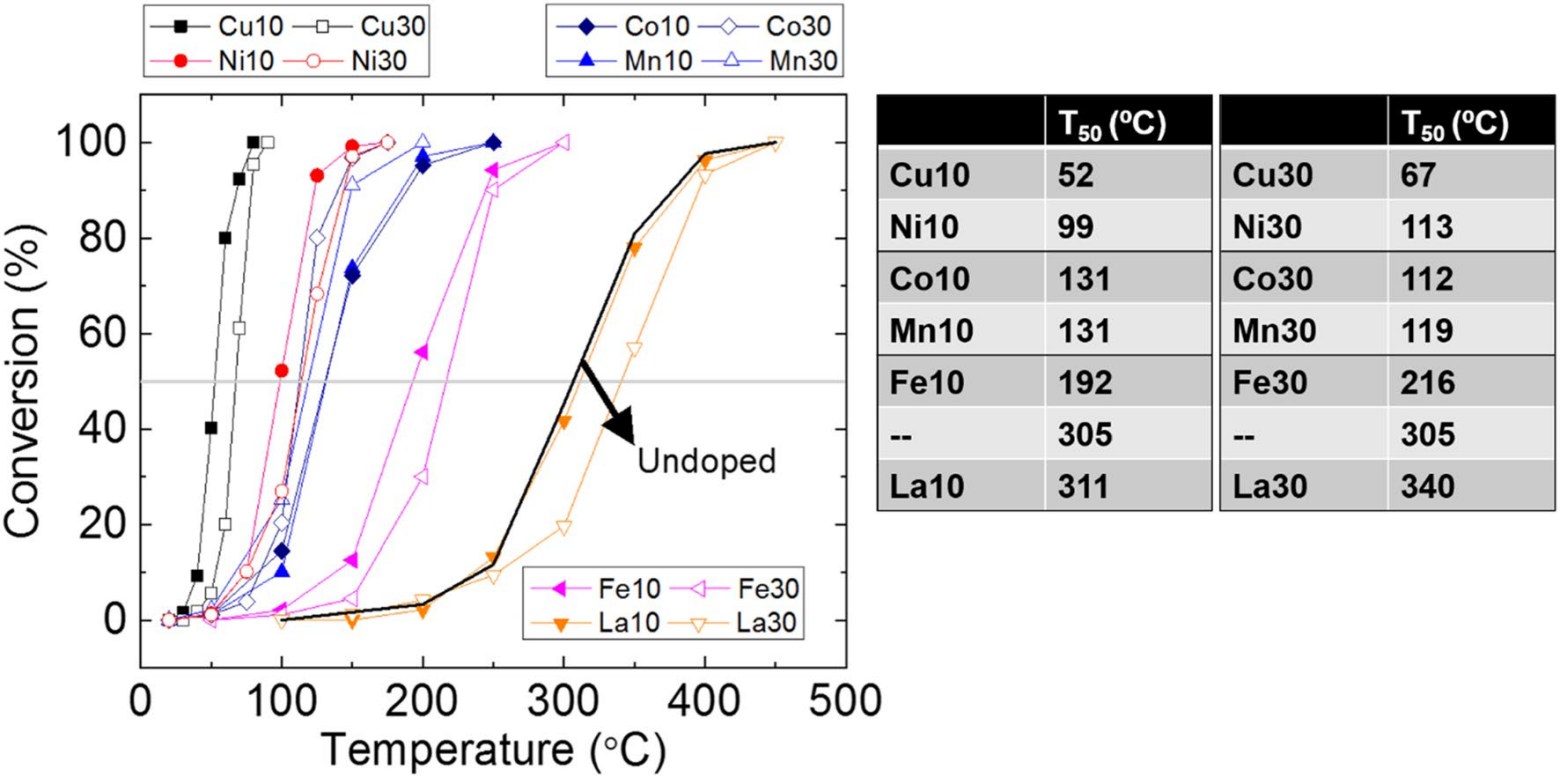
CO conversion as a function of temperature. Copper and nickel are the most effective dopants. T_50_ is the temperature for 50% conversion

The prominent impact of dopants on CO conversion is shown by conversion curves in Fig. 5. For all cases, high temperature gives higher conversion of CO; however, the required temperature to convert 50% of the CO varies greatly depending on what dopant is used. In general, high doping level lowered CO oxidation activity except cobalt and manganese. The overall behavior, high dopant concentration leading to lower CO conversion activity, suggests that not the absolute surface concentration of oxygen vacancy but the “readiness” of the doped cerium oxide to give away lattice oxygen governs the CO oxidation kinetics. The usual indicator found in the literature for this “readiness” is the oxygen vacancy formation energy. Theoretical calculation predicts smaller oxygen vacancy formation energy for copper (− 1.49 eV) and nickel (− 0.45 eV) than cobalt and manganese [39]. Cobalt and manganese had very close value around + 0.5 eV [39], and we see that the Co10 and Mn10 give almost the same conversion curves. This experiment-computation agreement for cobalt and manganese was less satisfactory for doped ceria nanorods from hydrothermal synthesis route [6]. We can also quickly find theoretical predictions that deviate from what we observe. Lanthanum dopant was the only one without recognizable beneficial impact on CO oxidation even though theoretical prediction suggests promoted CO adsorption on (111) and (110) ceria surfaces due to La doping [40]. Another theoretical prediction proposed similar oxygen vacancy formation energy for iron and copper [41], but these two dopants are very different in terms of observed catalytic activity. Based on these observations, it is not clear if the oxygen vacancy formation energy calculation is enough to predict CO oxidation catalytic activity of doped ceria catalysts. To date, CO adsorption/CO_2_ desorption energy calculation is rare for doped ceria catalysts. We think that more theoretical/experimental study is needed in this aspect. For experimental study, it is important to minimize microstructural change while varying the dopants [42]. We suggest that electrospinning can be a nice platform for this since the fiber diameter is determined by solvent evaporation and electrospinning condition.

**Fig. 6:**
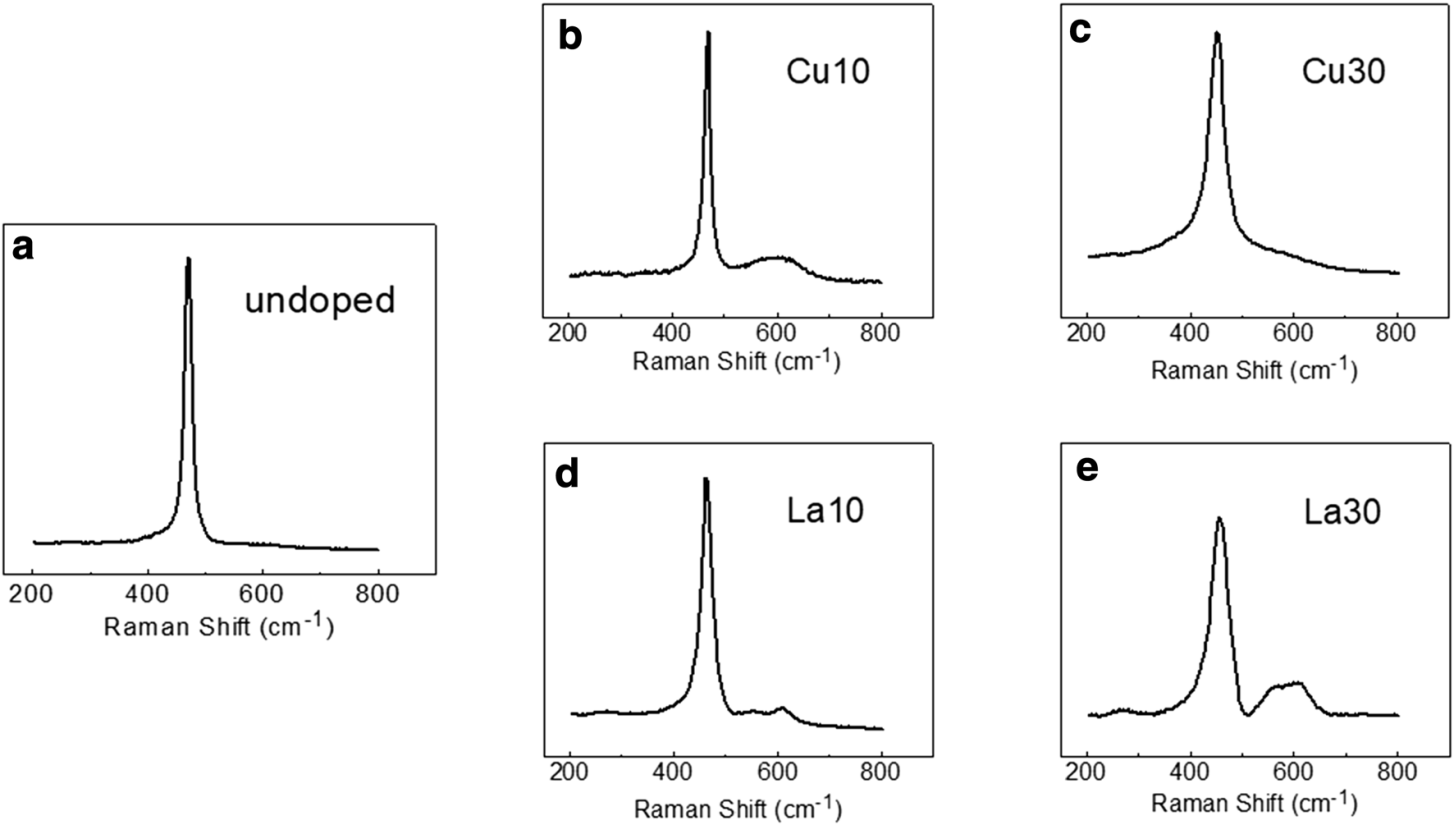
Raman spectra from as-calcined nanofiber samples

Figure 6 shows Raman features from the undoped ceria, the most active copper-doped ceria, and the least active lanthanum-doped ceria. The Cu30 sample has much weaker oxygen vacancy peak compared to Cu10 whereas higher lanthanum doping level gives higher concentration of the oxygen vacancy confirmed by the Raman spectrum (Fig. 6e). The La30 had the most prominent oxygen vacancy peak (~ 600 cm^−1^) and the largest unit cell volume (see Additional file 1) among all samples yet showing the worst activity toward CO oxidation. Reportedly, without using multiple wavelengths of laser, it is hard to make solid correlation between the observed Raman signal and the surface chemistry of ceria [43]. However, it is also known that surface is usually more reduced than bulk [25]. With observed strong oxygen vacancy peak, La30 sample clearly indicates that the concentration of the already-existing oxygen vacancy, when beyond certain level, is not the main factor that determines the CO oxidation kinetics of ceria-based catalysts. In this work, the identity of dopant dominates the CO oxidation catalysis over pre-existing oxygen vacancy concentration or electrospun fiber surface area. The weak dependence on surface area has been previously reported [42].

Copper and nickel are the most effective dopants for cerium oxide fibers in this study. Copper-doped cerium oxide has been studied intensively due to its high CO oxidation activity. Our result agrees with the literature showing great CO oxidation activity. The 50% conversion temperature (T_50_) for the 10 mol% copper is just 52 °C. For Cu-Ag co-doped ceria, T_50_ was previously reported to be 65 °C [39]. The same for the 10 mol% nickel is 99 °C in this work. For these two dopants, it has been suggested that the metal oxide cluster-ceria interface sites are the active sites [44, 45]. For nickel, this interfacial site concentration was shown to decrease when nickel doping level goes up from 5 mol% to 20 mol% for mesoporous ceria [45]. In this work, we were able to detect second phase CuO XRD peaks at 30 mol% doping. We can infer that the CuO_x_ cluster-ceria interfacial site concentration has started decreasing even before we reach 30 mol% doping level of copper leading to less active catalyst. Since copper doping was most effective for catalyzing CO oxidation, we listed previous reports on low temperature CO oxidation by copper-doped cerium oxide catalysts in Table 2.

**Table 2:** CO oxidation behavior of nanostructured, copper-doped ceria samples. T_50_ is the temperature for 50% conversion

Ceria morphology/copper mol%	Feed gas	Balance gas	T_50_	WHSV (mL h^−1^ g^−1^)	CO_2_ generation Rate at 50 °C (mol g^−1^ s^−1^)
Nanosheets/10 [29]	1% CO, 10% O_2_	N_2_	83 °C	60,000	1.7E−07
Nanoparticle/5 [39]	1% CO, 1% O_2_	Ar	65 °C	120,000	6.9E−07
Nanoparticle/30 [46]	1% CO, 0.6% O_2_	N_2_	74 °C	60,000	1.2E−08
Nanosphere/4 [33]	1% CO, 10% O_2_	N_2_	170 °C	96,000	0
Microscale particle/5 [47]	1% CO, 1% O_2_	N_2_	125 °C	9600	0
Nanoparticle/10 [48]	0.2% CO, 5% O_2_	Helium	59 °C	30,000	1.4E−06
Nanofiber/10 (this work)	1% CO, 2% O_2_	N_2_	52 °C	30,000	3.6E−07

*WHSV* weight hourly space velocity

From Fig. 5, we can see that only Co and Mn dopants deviate from the general trend of less activity with higher concentration (~ 30 mol%) of dopant. The general microstructural trend should remain the same for all dopants in this study as we increase the doping level: Interfacial site concentration on the solid surface increases initially upon doping but then decreases as doping level increases due to cluster size growth. The CO oxidation behavior of iron-doped ceria fiber follows the trend of copper and nickel suggesting that the interfacial sites being active. For cobalt 30 mol% doping, we speculate that additional CO oxidation activity from the XRD-confirmed Co_3_O_4_ second phase leads to eventually higher CO conversion compared to 10 mol% cobalt. Co_3_O_4_ can be very active towards CO oxidation by itself [49]. For manganese 30 mol% doped case, no second phase was detected from the XRD. However, it is still possible that we have small Mn_x_O_y_ islands on the ceria surface and they are active like Co_3_O_4_ providing additional active sites. For manganese, this high doping level was previously proven to be effective for CO oxidation [50].

**Fig. 7:**
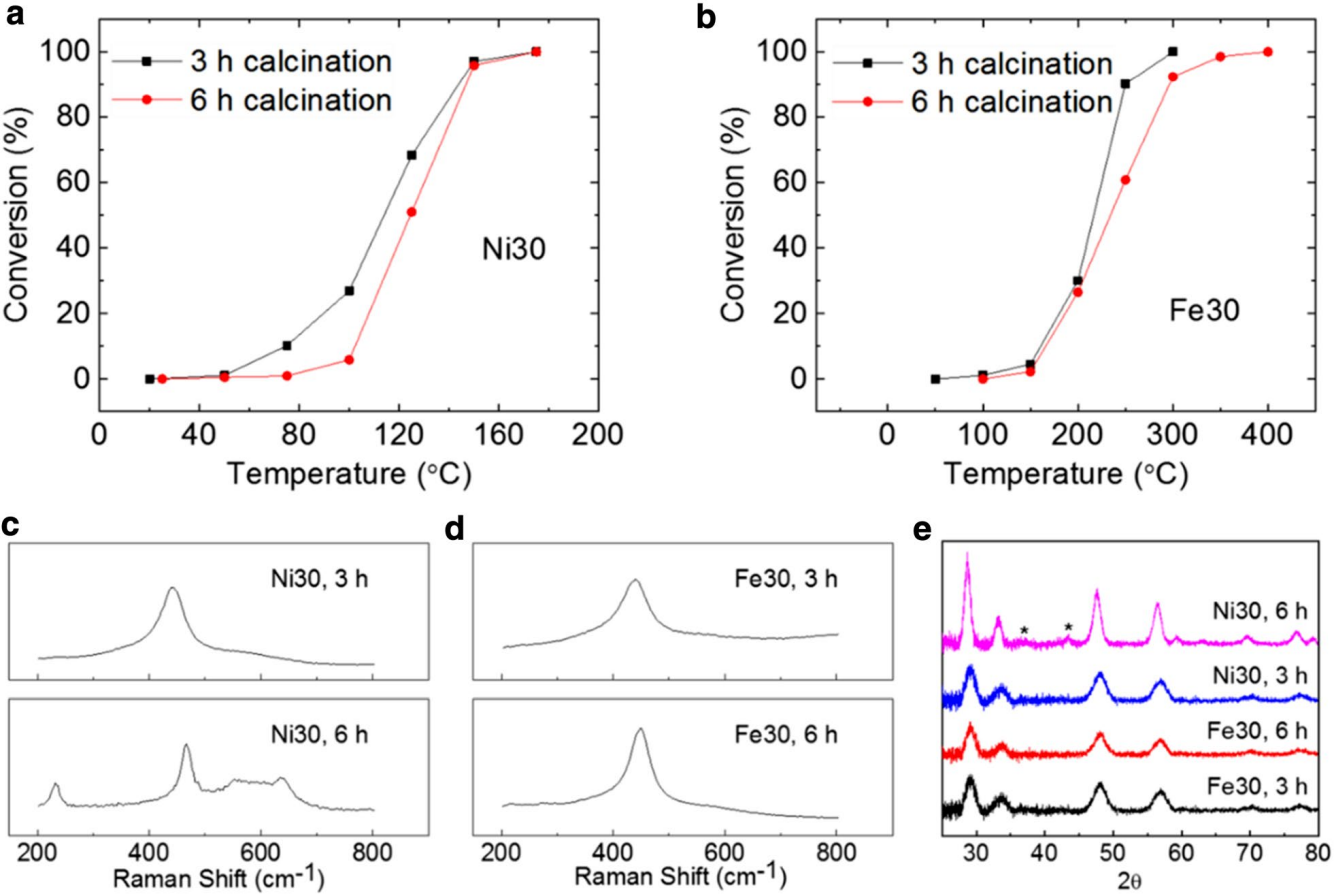
CO conversion change by longer calcination time for **a** Ni30 and **b** Fe30. **c**, **d** Raman spectra from tested samples. **e** X-ray diffraction patterns from tested samples. NiO peaks are marked for the 6 h calcinated Ni30 pattern

Fiber microstructural change impacts CO oxidation differently depending on what dopant is employed. To test the sensitivity of the CO oxidation kinetics, we prepared 6 h calcinated samples for Fe30 and Ni30. Figure 7a, b show conversion curves from samples with two different calcination times. By simply holding the temperature longer at 600 °C, we were able to induce different degree of microstructure change to the doped fibers under the accelerated degradation condition. Ni30 went through more significant changes than Fe30 as evidenced by Raman spectra (Fig. 7c, d) and X-ray diffraction patterns (Fig. 7e). The newly emerged Raman peaks in Fig. 7c for Ni30 6 h sample were reported previously in relation to aliovalent doping and oxygen vacancies [51] rather than NiO islands. The inherent advantage of nickel doping over iron doping is obvious as seen in Fig. 7a, b. Even with severe adverse microstructural change that lowers CO conversion for all temperatures below 150 °C, the temperature of 100% conversion did not change for Ni30 after longer calcination at 600 °C. For Fe30, longer calcination clearly raised the temperature of 100% conversion by 100 degrees even when changes in Raman spectrum and XRD pattern are relatively small compared to Ni30.

**Fig. 8:**
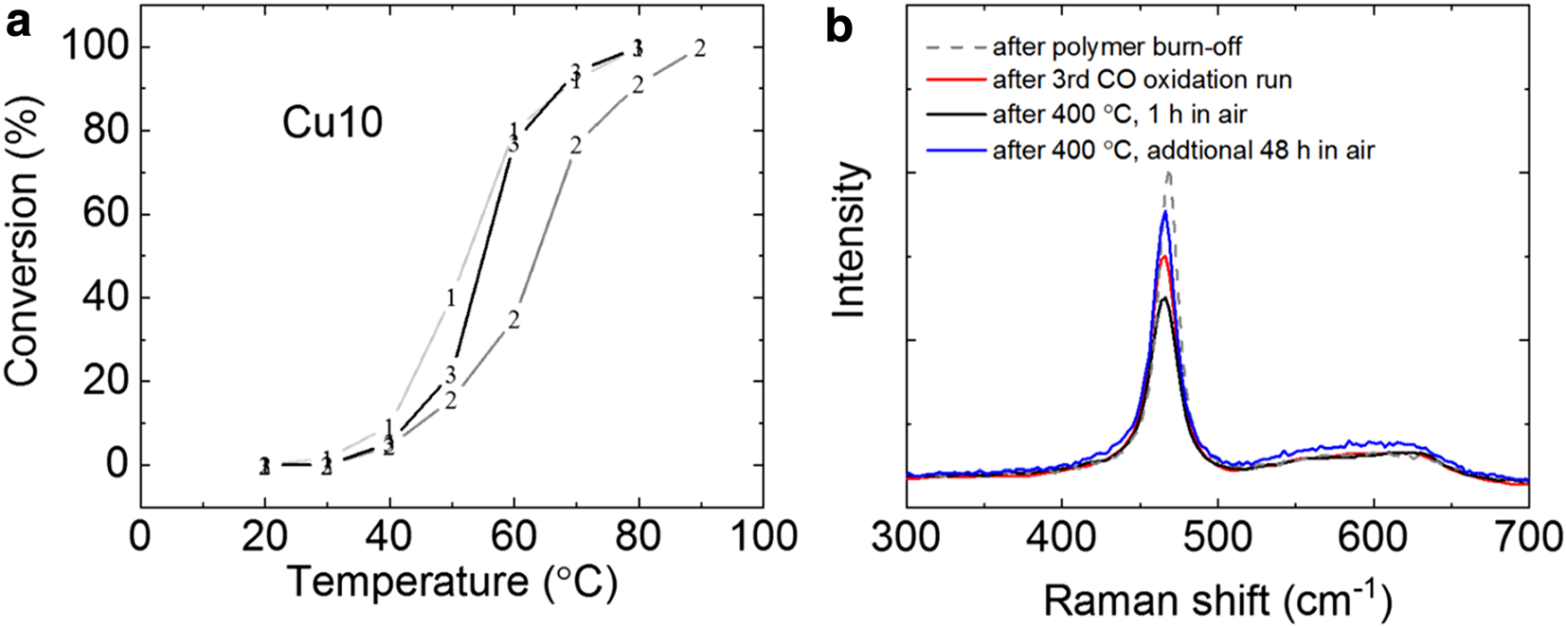
**a** CO conversion change over repeated oxidation runs on the same exact catalyst. The numbers show the sequence of the data collection. All conversion data were collected during heating up. **b** Raman spectra from the exact same sample in panel a)

Durability of the ceria fiber catalyst is tested by sequential CO oxidation runs. Figure 8a shows conversion curves of one exact Cu10 sample. After reaching the temperature for 100% conversion, 80 °C, of the first CO oxidation run (1, light gray), the sample was cooled down in nitrogen flow. Second oxidation run starting again from room temperature showed lower conversion. The T_50_ got raised by 10 degrees, which is a significant change considering the results in Fig. 5. We had to go to 90 °C to achieve 100% conversion this time (2, dark gray). We then heated up the same Cu10 to 400 °C in air. This treatment was effective in partially reviving the catalytic activity as seen in Fig. 8a (3, black). At 50 °C, the air-annealed fiber already shows higher conversion than the second curve. From 60 °C on, the conversion is recovered to the original value. It is encouraging that we were able to recover the conversion by simple heat treatment in air even though it is surprising to see the dynamic change during these three consecutive runs. Previously, beneficial impact of nitrogen or hydrogen annealing at 450 °C was reported for low temperature (200 °C) processed ceria catalysts [21]. However, our electrospun samples had been heated to 600 °C in air in the fabrication step. Figure 8b shows Raman spectra of the same exact Cu10 sample. The dashed gray trace is from the sample right after calcination. This is before any CO oxidation test. After all the CO oxidation runs in Fig. 8a, the red spectrum is collected. We heated up the sample in air to 400 °C for 1 h duration (black trace) and additional 48 h duration (blue trace). We can see that the re-filling of oxygen vacancy to the level of the as-calcined state is surprisingly sluggish at this relatively high temperature compared to CO oxidation temperature. The oxygen vacancy re-filling is not complete even after 48 h based on the Raman spectrum while the Cu10 sample is the most active one among all tested cases. We again observe that oxygen vacancy detected from Raman spectroscopy cannot directly tell how high or low the CO oxidation activity would be. This may come from the fact that Raman spectroscopy gives too much of the bulk information rather than surface-specific characteristics when we use 514.5 nm laser [43]. We think that the regeneration by 400 °C air annealing in Fig. 8a should have the highest impact at the outer surface in the form of surface reconstruction. Copper oxide cluster-ceria interface sites are known to be active [44], and these sites should have been regenerated by the annealing, probably through re-dispersion of copper oxide clusters over the cerium oxide surface.

## Conclusion

We demonstrated low temperature CO oxidation using metal-free electrospun ceria catalyst with a variety of dopants. Even with high processing temperature to burn off polymeric constituent, the resulting ceria catalyst was still effective in low temperature CO oxidation. Especially, 10 mol% copper-doped ceria shows 50% conversion at just 52 °C with CO_2_ generation rate of 3.6 $\times$ 10^−7^ mol g^−1^ s^−1^ at 50 °C. XRD-detectable bulk second phase formation can be beneficial (e.g. cobalt) or detrimental (e.g. copper) depending on dopants. The identity of dopant dominates the CO oxidation catalysis over nominal oxygen vacancy concentration determined by dopant concentration or electrospun fiber surface area. Recently reported theoretical oxygen vacancy formation energy agrees with our catalytic activity results for copper, nickel, cobalt, and manganese dopants [39]. However, it is still possible that the doping-induced change in CO/CO_2_ molecule-cerium oxide surface binding energy determines the low temperature CO oxidation. Reactivation of the most active 10 mol% copper-doped ceria has been demonstrated by simple annealing in ambient air. The conventional practice of correlating oxygen vacancy detected by Raman spectroscopy to the CO oxidation activity should be reconsidered.

## Supplementary information

**Additional file 1. Figure S1.** Electrospinning setup. **Figure S2.** Fe-doped ceria fiber before polymer burn-off (left) and after (right). The polymer-free iron-doped ceria fiber sample (right, Fe30) was one of the many samples used for CO oxidation. **Figure S3.** a) Undoped ceria before polymer burn-off. b) Co30 sample after polymer burn-off and die pressing. This is the sample microstructure that goes into the CO oxidation reactor. Due to the brittleness of the ceria fiber, long fibers break down to be short fibers. We do not see round-shape particles.

## Data Availability

The datasets used and/or analyzed during the current study are available from the corresponding author on reasonable request.

## Abbreviations

BET: Brunauer–Emmett–Teller
MW: Molecular weight
TEM: Transmission electron microscopy
XRD: X-ray diffraction
YSZ: Yttria-stabilized Zirconia
