# Supplementary material for: Low temperature CO oxidation by doped cerium oxide electrospun fibers
Source: Nano Converg. 2020 Jun 29;7:22. doi: 10.1186/s40580-020-00234-7 (PMC7324448; doi:10.1186/s40580-020-00234-7)
Supplement: Supplementary file 1 — Additional file 1. Figure S1. Electrospinning setup. Figure S2. Fe-doped ceria fiber before polymer burn-off (left) and after (right). The polymer-free iron-doped ceria fiber sample (right, Fe30) was one of the many samples used for CO oxidation. Figure S3. a) Undoped ceria before polymer burn-off. b) Co30 sample after polymer burn-off and die pressing. This is the sample microstructure that goes into the CO oxidation reactor. Due to the brittleness of the ceria fiber, long fibers break down to be short fibers. We do not see round-shape particles. [file 40580_2020_234_MOESM1_ESM.docx]

**Additional Information for**

**Low temperature CO oxidation by doped cerium oxide electrospun fibers**

Myeongseok Sim^1^, Buhua Wang^2^, and Tae-Sik Oh*^1^

^1^Department of Chemical Engineering, Auburn University, Auburn, AL 36849, USA

^2^Department of Chemistry and Biochemistry, Auburn University, Auburn, AL 36849, USA

**taesik.oh@auburn.edu**

1. Electrospinning setup image.


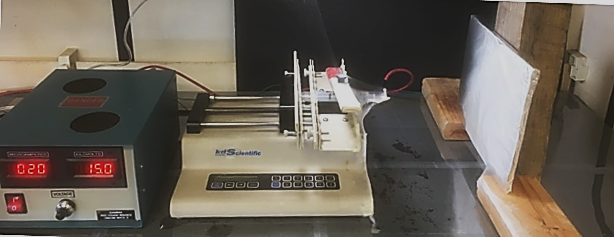


Figure S1. Electrospinning setup.

2. Sample image.

Figure S2. Fe-doped ceria fiber before polymer burn-off (left) and after (right). The polymer-free iron-doped ceria fiber sample (right, Fe30) was one of the many samples used for CO oxidation.

3. Lattice parameters and crystallite sizes from Rietveld refinement (samples in Figure 5)

|  | Lattice parameter (angstrom) | Crystallite size (nm) |  | Lattice parameter (angstrom) | Crystallite size (nm) |
| --- | --- | --- | --- | --- | --- |
| Cu10 | 5.424 | 11.6 | Cu30 | 5.417 | 15.5 |
| Ni10 | 5.413 | 10.3 | Ni30 | 5.418 | 10.4 |
| Co10 | 5.417 | 13.4 | Co30 | 5.414 | 14.4 |
| Mn10 | 5.404 | 10.1 | Mn30 | 5.392 | 6.7 |
| Fe10 | 5.410 | 9.7 | Fe30 | 5.380 | 5.4 |
| Undoped | 5.418 | 15.4 | -- | -- | -- |
| La10 | 5.448 | 12.4 | La30 | 5.489 | 7.3 |

4. SEM images


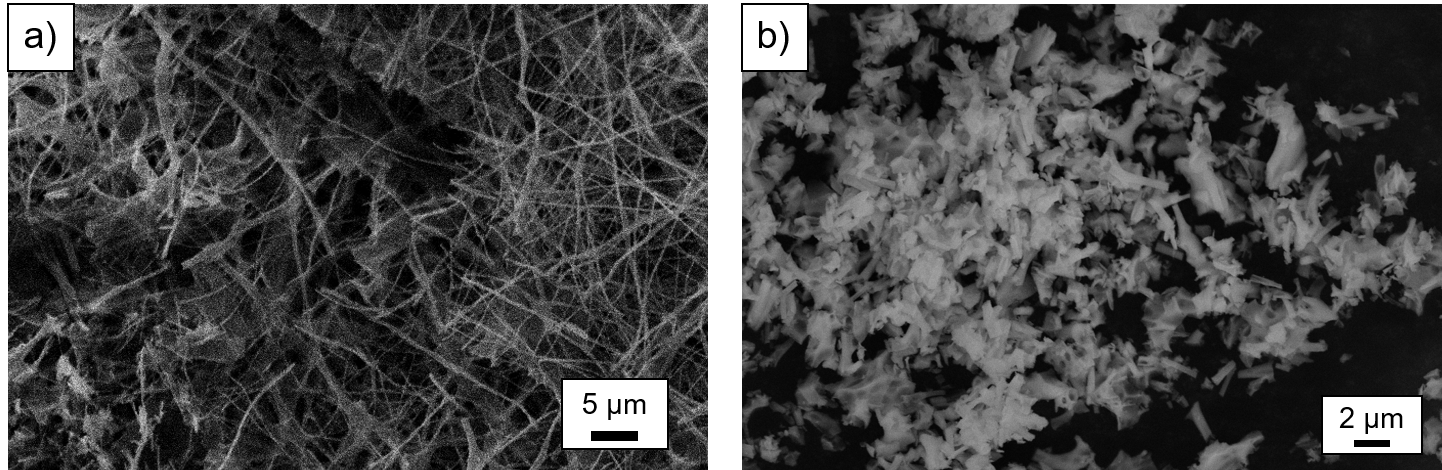


Figure S3. a) undoped ceria before polymer burn-off. B) Co30 sample after polymer burn-off and die pressing. This is the sample microstructure that goes into the CO oxidation reactor. Due to the brittleness of the ceria fiber, long fibers break down to be short fibers. We do not see round-shape particles.
